# Behaviour change techniques, intervention features and usability of diet apps

**DOI:** 10.1016/j.pmedr.2025.103085

**Published:** 2025-05-02

**Authors:** Richard Pavlicek, Kevin A. Cradock

**Affiliations:** a Department of Health and Nutrition Science, Atlantic Technological University, Ash Lane, Sligo, Ireland

**Keywords:** Behaviour change techniques, Intervention features, Usability, Diet apps

## Abstract

**Objective:**

Identify the behaviour change techniques and intervention features in popular diet apps.

**Methods:**

The most popular diet apps were identified from the top 200 ranked apps in the Health & Fitness sections of the App Store and Google Play in September 2023. Selected apps were used for one week and their content analysed for the presence of behaviour change techniques and intervention features. Apps were rated using the Mobile App Rating Scale score.

**Results:**

Thirteen apps with 23 app versions (free & premium) were included. The mean number of behaviour change techniques was 18.3 ± 5.8. The most frequently coded behaviour change techniques were predominantly from the ‘Goals and planning’ and ‘Feedback and monitoring’ categories. Apps contained 21.1 ± 6.1 intervention features and scored a mean Mobile App Rating Scale rating of 3.8 ± 0.3. There was a strong, statistically significant correlation (*r* = 0.69; *p* = 0.01) between the number of behaviour change techniques and the Mobile App Rating Scale rating. Analysis identified discrepancies between the Mobile App Rating Scale rating and the App Store and Google Play ranking systems.

**Conclusions:**

Selected apps contained a high number of behaviour change techniques and intervention features. Most included apps lacked an evidence base and safety features. App engagement, optimal use of time, safety features and app ranking systems require further research to provide evidence-based recommendations.

## Highlights

• Diet apps had a high number of behaviour change techniques, intervention features.
• Included diet apps lack some important behaviour change techniques.
• Diet app ranking systems require greater transparency.
• Future studies should look at how users engage with diet apps.
• Most included diet apps lacked an evidence base and safety features.

## 1 Introduction

Changing diet behaviour is recommended for disease prevention, management and healthy lifestyle adoption (Ford et al., 2012) but changing diet behaviour is complex. Social, physical, environmental, neuroendocrine, and genetic factors influence diet behaviour change (Story et al., 2008). With numerous barriers, including stress, work, increasing food costs, food availability, climate and culture, it is no surprise that most people fail to initiate or maintain healthy diet behaviours (Middleton et al., 2015) with only 20 % succeeding in maintaining weight-loss long-term (Rosenbaum and Foster, 2023). Diet behaviour interventions are typically applied individually or in groups, either face-to-face or online.

Digital and online delivery of interventions have become increasingly popular due to convenience, scalability, lower cost and user-friendly interfaces (van Genugten et al., 2016). Furthermore, digital delivery modes can facilitate barrier identification, problem-solving (van Genugten et al., 2016) via personalisation, even in large-scale interventions (Teixeira and Marques, 2018) and have also been shown to be cost effective in chronic disease treatment (Kyaw et al., 2023). In obese adolescents, mobile apps are more cost-effective than traditional interventions and offer higher retention rates with comparable clinical outcomes (Vidmar et al., 2018). Mobile applications are scalable and can deliver interventions irrespective of time and place, potentially reaching inaccessible individuals (Klasnja and Pratt, 2012). Mobile applications use behaviour change theories and techniques to facilitate behaviour change.

Theory provides a foundation for interventions and helps identify relevant behaviour change techniques (BCTs) linked to theoretical constructs (Timlin et al., 2020). BCTs are the smallest observable and replicable “active ingredients” for designing interventions (Michie and Johnston, 2012) and evidence links better diet behaviour outcomes with more BCTs (Lara et al., 2014), but others show no association (Dombrowski et al., 2012). The lack of clarity around the optimal number of BCTs may stem from varying engagement and intervention fidelity (Bellg et al., 2004).

National Institute for Health and Care Excellence (NICE) guidelines for new technologies (National Institute for Health and Care Excellence, 2014) recommend using BCTs from the ‘Goals and planning’ and ‘Feedback and monitoring’ categories in dietary interventions. Michie et al (Michie et al., 2009). suggested that ‘Self-monitoring’ would likely be effective in healthy eating interventions, also highlighted where diet app users who frequently monitored their meals, body weight, and exercise exhibited significant weight loss over time (Han and Rhee, 2021).

Other BCTs linked to dietary behaviour change that could have a beneficial effect include ‘Intention formation’, ‘Specific goal setting’, ‘Review of behavioural goals’ and ‘Feedback on performance’. (Direito et al., 2014) In weight-loss research, the BCTs ‘Social support (unspecified)’, ‘Self-monitoring of behaviour’ and ‘Goal-setting (outcome)’ were considered effective (Ashton et al., 2020). Even though BCTs can improve diet and health-related outcomes (Direito et al., 2014), their use in commercial apps is relatively sparse. The absence of evidence-based BCTs in apps might lead to inferior outcomes compared to face-to-face interventions, where effective techniques have been identified (Lewis et al., 2016a). In addition to BCTs, apps contain other features impacting user engagement.

Mobile app features can potentially enhance user care and intervention outcomes. Apps can include input features such as recording of weight or dietary intake, while output features can include macronutrient analysis or reports (Franco et al., 2016). Online diet interventions require users to engage with the app for it to work (Freyne et al., 2017), with engagement typically encouraged using push notifications or gamification (Looyestyn et al., 2017). Daily diet monitoring can be time and labour-intensive for the user, therefore, some apps include features such as photo or audio logging (Schumer et al., 2018). Identifying frequently used intervention features may help the design and implementation of future app development.

Over 60 % of the world's population own a smartphone in 2024, indicating widespread availability and use of apps. In addition, over 60 % of the world's population are either overweight or obese, highlighting a pressing need for diet behaviour change. There is a lack of regulation, information and clarity on the content, source of information and techniques used to facilitate behaviour change in these apps. This study seeks to identify and analyse the most popular diet apps available in the Health & Fitness section of the App Store and Google Play. This study aims to provide a systematic content analysis of identified apps' BCTs, intervention features and app quality using the Mobile App Rating Scale (MARS).

## 2 Methods

### 2.1 App selection

Provisional searches were conducted in the 200 top-ranked apps in Medical; Education; Food and Drink; and Health and Fitness categories of the App Store and Google Play. Only the Health and Fitness section was found to contain diet apps with the potential to match the selection criteria. This study focused on the free and premium apps (additional cost) available in the free category. The Health & Fitness section was accessed on 28th September 2023, and identified apps were recorded. The App Store and Google Play were accessed from Ireland on iPhone 13, iOS 16.6 and Huawei ALE-L21, Android 6.0. Smartphones were selected for this purpose, as the App Store and Google Play offer only a limited range of apps available for tablets and laptops.

### 2.2 Inclusion and exclusion criteria

Inclusion criteria for selection from the top 200 list focused on adults aiming to change their diet behaviour through diet tracking or monitoring, meal planning, intermittent fasting or other specific diet approaches. Only apps in the English language were included. Exclusion criteria removed apps focused on shopping, recipes, food delivery or other non-diet-related behaviours. Diet apps focusing on a narrow population group (e.g., type 1 diabetes, cancer, heart disease or pregnancy-specific) or behaviours such as alcohol or water intake only were also excluded. Apps including water, alcohol or physical activity interventions were included only if the diet component was also present. The user rating of the app was not used as a selection criterion, as it does not necessarily reflect the app's quality (Stoyanov et al., 2015). No ethical approval was required as this study was a content analysis of publicly available apps and no individual or personal data were used for analysis.

### 2.3 Data extraction

Both authors downloaded all apps' free and premium (paid) versions that met the inclusion criteria. Both authors independently coded all apps for BCTs, intervention features, and MARS ratings. The testing and coding were conducted from October 2023 to March 2024. Each app was tested for one week in its free and premium versions, if available. Data were compiled using Microsoft® Excel for Mac (Version 16.83; Microsoft Corp., Redmond, Washington, United States of America).

### 2.4 Behaviour change techniques assessment

The 93-item BCT taxonomy version 1 by Michie et al (Michie et al., 2013). was used for BCT coding. To improve confidence in the BCT coding process and inter-rater reliability, both reviewers completed BCT taxonomy version 1 training and achieved >80 % in coding training. The BCTs were entered into an Excel checklist, and coding was accompanied by a screenshot containing the BCT to provide an audit trail for both reviewers to check the coding accuracy. For a BCT to be coded as present, the following conditions were required: a) only BCTs related to diet behaviour were coded, b) the presence of a BCT could not be inferred and must match the BCT description, c) features present outside of the app environment (e.g. external link to app website) were not coded for BCTs and d) when calculating the total number of BCTs per app, each BCT was only counted once, even if present in the app on multiple occasions. One week was spent coding each app version during the initial coding process. Each week, the coding of the coded app was discussed, and disagreements were solved via discussion. BCTs coded for free versions were assumed to be present in the premium ones. A minimum of 70 % coding agreement was required, which was considered an acceptable inter-rater reliability in another study (Antezana et al., 2020). The entire process was repeated for app versions not meeting the >70 % agreement minimum. The Noom app was used for two weeks by one author during the initial coding process due to technical difficulties requiring communication with the app's support team.

### 2.5 Intervention features assessment

Based on previous studies analysing mobile app features, a list of 34 intervention features was created and divided into five categories: Healthy eating advice, scientific basis and safety; Food; Tracking and goal setting; Social; Technology. The rationale for and references linked to each of the intervention features is documented in Appendix 1. Both authors independently coded all free and premium app versions similar to the BCT approach and recoded if inter-rater reliability was <70 %.

### 2.6 Quality assessment

The app quality testing was conducted using the MARS scale (Stoyanov et al., 2015), which provides an overall usability rating and four separate usability domains, which include eighteen items. Each item is rated on a 5-point scale, from one inadequate to five excellent. Before rating, the authors watched the MARS training video to improve reliability. The two authors scored the MARS items independently, and the final score was calculated using an average of both authors' coding.

### 2.7 Analysis

Descriptive statistics were used to describe the number of BCTs and intervention features for included apps in terms of means and standard deviations. Normality was assessed using the Shapiro-Wilks test. Where data were normally distributed, a Pearson product correlation was used. Where data were not normally distributed, a Spearman's rank correlation test was used. Statistical significance was calculated using the T-statistic and was set at *p* ≤ 0.05. As the App Store and Google Play rankings are reported in hierarchical order, the Spearman's rank correlation test was used to assess the relationship between the apps' ranking and BCTs, MARS and intervention features. Data were analysed using Microsoft® Excel for Mac (Version 16.83; Microsoft Corp., Redmond, Washington, United States of America).

## 3 Results

### 3.1 Selection summary

Thirteen apps (Body Fast; Calorie Counter +; Cronometer; Fast Easy; Fastic: Intermittent Fasting; Fat Secret; Lose It!; Macros; My Fitness Pal; My Net Diary; Noom; Yazio; Zero) were selected for analysis. All apps offered a free and premium version except for Zero, Noom and FastEasy, which only offered a paid premium version. Twenty-three versions of apps were selected for coding.

### 3.2 Behaviour change techniques

Inter-rater reliability of BCT coding was classified as ‘Strong’ (κ = 0.87). The 45 BCTs identified are documented in Table 1. Forty-eight BCTs were not coded. The mean number of BCTs per app version was 18.3 ± 5.8. As no free versions of the Fast Easy, Noom and Zero apps were available, no BCTs were coded. Free app versions included a mean of 17.0 ± 4.2 BCTs, with Fastic Intermittent Fasting containing the most (*n* = 23) and Fat Secret the least (*n* = 11) as documented in Table 2. Premium-app versions included a mean of 19.4 ± 6.6 BCTs, with Noom containing the most (*n* = 33) and Macros the least (*n* = 13). All 16 categories of BCTs except ‘Scheduled consequences’ were represented.

**Table 1:** Frequency of behaviour change techniques in top-rated diet apps available in google play and app store in Ireland, 2023.

		**Free**		**Premium**	
**BCT Number**	**BCT Label**	**n**	**%**	**n**	**%**
**The most frequently used BCTs**					
1.1	Goal setting (behaviour)	9	90	13	100.0
1.3	Goal setting (outcome)	9	90	13	100.0
1.4	Action planning	9	90	13	100.0
2.2	Feedback on behaviour	9	90	13	100.0
2.3	Self-monitoring of behaviour	9	90	13	100.0
2.4	Self-monitoring of outcome(s) of behaviour	9	90	13	100.0
7.1	Prompts/cues	8	80	13	100.0
2.7	Feedback on outcome(s) of behaviour	8	80	12	92.3
9.1	Credible source	8	80	12	92.3
4.1	Instruction on how to perform a behaviour	7	70	11	84.6
**Frequently used BCTs**					
10.3	Non-specific reward	7	70	10	76.9
10.10	Reward (outcome)	6	60	10	76.9
3.1	Social support (unspecified)	5	50	9	69.2
5.1	Information about health consequences	5	50	9	69.2
8.1	Behavioural practice/rehearsal	6	60	9	69.2
8.3	Habit formation	6	60	9	69.2
1.6	Discrepancy between current behaviour and goal	5	50	7	53.8
12.4	Distraction	2	20	6	46.2
1.5	Review behaviour goal(s)	3	30	5	38.5
6.1	Demonstration of the behaviour	4	40	5	38.5
10.9	Self-reward	2	20	5	38.5
1.2	Problem solving	1	10	4	30.8
5.4	Monitoring of emotional consequences	2	20	4	30.8
6.3	Information about others' approval	3	30	4	30.8
8.2	Behaviour substitution	3	30	4	30.8
10.6	Non-specific incentive	2	20	4	30.8
11.2	Reduce negative emotions	0	0	4	30.8
1.7	Review outcome goal(s)	2	20	3	23.1
5.3	Information about social and environmental consequences	0	0	3	23.1
6.2	Social comparison	1	10	3	23.1
12.1	Restructuring the physical environment	0	0	3	23.1
**Rarely used BCTs**					
1.9	Commitment	1	10	2	15.4
8.6	Generalisation of a target behaviour	1	10	2	15.4
10.8	Incentive (outcome)	2	20	2	15.4
13.2	Framing/reframing	1	10	2	15.4
1.8	Behavioural contract	1	10	1	7.7
3.2	Social support (practical)	0	0	1	7.7
4.2	Information about antecedents	0	0	1	7.7
5.6	Information about emotional consequences	0	0	1	7.7
7.7	Exposure	0	0	1	7.7
8.4	Habit reversal	1	10	1	7.7
8.7	Graded tasks	0	0	1	7.7
9.2	Pros and cons	0	0	1	7.7
15.1	Verbal persuasion about capability	0	0	1	7.7
15.3	Focus on past success	0	0	1	7.7

Note: Behaviour Change Technique (BCT) number and label are documented according to the version 1 taxonomy by Michie et al (Milne-Ives et al., 2023). BCTs and how frequently they were coded are documented for both Free and Premium app versions.

**Table 2:** Ranking, Mobile App Rating Scale, Behaviour Change Techniques and Intervention Features of Top-Rated Diet Apps in Google Play and App Store in Ireland, 2023.

**App**	**App Store Ranking (0−200)**	**Google Play Ranking (0–200)**	**MARS**	**Number of BCTs (Free)**	**Number of BCTs (Premium)**	**Number of Intervention Features (Free)**	**Number of Intervention Features (Premium)**
MyFitnessPal	2	9	4	22	30	23	27
Calorie Counter +	13	5	3.4	15	15	27	28
MyNetDiary	40	48	4.2	20	21	26	30
Lose It!	53	120	4	16	16	21	25
Fastic	58	55	3.9	23	27	19	24
Cronometer	62	114	4.2	16	18	27	29
BodyFast	71	35	3.9	22	25	12	13
FastEasy	80	160	3.5	N/A	14	N/A	11
Zero	85	159	3.8	N/A	18	N/A	12
Stupid Simple Macro Tracker	90	96	3.1	12	13	15	17
Noom	109	93	4.3	N/A	33	N/A	29
YAZIO	172	163	4	18	21	25	25
FatSecret	198	139	3.4	11	13	20	23

Note: App Store and Google Play rankings, Mobile App Rating Scale (MARS), number of behaviour change techniques (BCTs) and intervention features are included for Free and Premium app versions.

### 3.3 Intervention features

All app versions were assessed for 34 possible intervention features (Table 3 ), with an average of 21.1 ± 6.1. At least one more intervention feature was identified in the premium compared to the free versions in all but one app (Yazio). The free app versions included a mean of 20.9 ± 5.1 (range 12–27) features with Calorie Counter + and Cronometer (*n* = 27) containing most and BodyFast least (*n* = 12). The features ‘Diet plan’ and ‘Chatbot’ were absent in all free app versions. The premium app versions included a mean of 21.4 ± 6.9 (range 11–30) intervention features with MyNetDiary (*n* = 30), Cronometer (*n* = 29) and Noom (n = 29) containing most and FastEasy least (*n* = 11). ‘Weight monitoring/tracking’ and ‘Weight progress graphs or charts’ were features present in all premium app versions (*n* = 13).

**Table 3:** The Frequency of Intervention Features in Top-Rated Diet Apps in Google Play and App Store in Ireland, 2023.

	**Free**		**Premium**	
**Intervention Feature**	**n**	**%**	**n**	**%**
**Healthy eating advice, scientific basis, and safety**				
Educational, health and lifestyle information	8	80	12	92.3
Recommended water consumption	5	50	11	84.6
Energy requirement calculation	9	90	10	76.9
Safety net on maximal weight loss goal	6	60	7	53.8
BMI use	5	50	6	46.2
Safety net on weight loss that can be achieved	3	30	4	30.8
**Food**				
Recipes	6	60	11	84.6
Add new food and remember favourite foods	9	90	10	76.9
Calories by meal	9	90	10	76.9
Food database	9	90	10	76.9
Macronutrient intake – range or amount, absolute, g/kg/bw number	8	80	10	76.9
Macronutrient intake - distribution (%)	7	70	7	53.8
Recommends intake or limits other nutrients (i.e., saturated fat, fibre, salt, and sugar)	4	40	7	53.8
Pictures of food items	4	40	5	38.5
Diet plan	0	0	5	38.5
Shopping list	2	20	2	15.4
**Tracking, goal setting**				
Weight monitoring/tracking	10	100	13	100
Weight progress graphs or charts	10	100	13	100
Diet tracking	9	90	12	92.3
Activity tracking	8	80	11	84.6
Weight target date (direct or indirect – set kg to lose per week)	9	90	11	84.6
Energy intake / macronutrient graphs or charts	8	80	10	76.9
circumference measurements (waist, hips or neck)	4	40	6	46.2
Monitoring / tracking of emotions, negative thoughts, stress	2	20	6	46.2
**Social**				
Blog: written by app	9	90	11	84.6
Social networking and support: sharing on forum, messages to other users within app	8	80	10	76.9
Social media sharing; link to social media apps	8	80	9	69.2
Sharing with professionals or online coaching option	5	50	7	53.8
**Technology**				
Combination with other devices (wearable tracker, website)	7	70	11	84.6
Barcode scanner	8	80	10	76.9
Gamification	8	80	10	76.9
Ability to export data/details about meals/daily summaries	6	60	9	69.2
Camera or audio diet logging	2	20	5	38.5
Chatbot: ability to communicate with Artificial Intelligence feature	0	0	2	15.4

Note: The frequency of intervention features in the free and premium versions of included apps.

### 3.4 MARS rating

Apps (n = 13) reached a mean MARS score of 3.8 ± 0.4 (range 3.1–4.3; Table 2). Apps performed worst in the Information subsection with a mean score of 3.4 ± 0.3 and best in Functionality with a mean score of 4.2 ± 0.4. Engagement and Aesthetics were 3.8 ± 0.5 and 3.9 ± 0.5 respectively. All thirteen apps were considered high quality, reaching an overall mean score of at least 3.0. Item 19, ‘Evidence base’ from the Information domain, was omitted as no research studies were available for most apps.

### 3.5 Correlation analysis

The App Store and Google Play rankings for the included apps are documented in Table 2. Apps are represented from the highest to the lowest ranked according to the App Store and compared to their MARS score. Initial analysis showed strong correlations between the MARS score and the number of BCTs (*r* = 0.69), Intervention features (r = 0.69), both statistically significant (Table 4). Further analysis revealed no statistically significant relationship between the App Store or Google Play rankings and the MARS score, number of BCTs or intervention features. Subgroup analysis of BCT categories showed statistically significant strong correlations between MARS score and the ‘Social support’ (*r* = 0.77), ‘Shaping knowledge’ (*r* = 0.72) and ‘Comparison of behaviour’ (*r* = 0.67) categories. Similar analysis of intervention features categories showed a statistically significant correlation between MARS score and ‘Social’ (*r* = 0.62) category.

**Table 4:** Correlation Analysis of Top-Rated Diet Apps in Google Play and App Store in Ireland (Premium Versions), 2023.

	**Correlation (r)**	***p* value (2-tailed)**
MARS and BCT	0.69*	0.01
MARS and Intervention features	0.69	0.01
BCT and Intervention features	0.40	0.17
**App Store**		
Rank and MARS	−0.19	0.53
Rank and BCT	−0.25	0.41
Rank and Intervention features	−0.39	0.18
**Google Play**		
Rank and MARS	−0.09	0.78
Rank and BCT	−0.39	0.18
Rank and Intervention features	−0.44	0.13
**BCT Categories**		
MARS and ‘Goals and planning’ BCTs	0.54*	0.06
MARS and ‘Feedback and monitoring’ BCTs	0.35	0.24
MARS and ‘Social support’ BCTs	0.77	< 0.01
MARS and ‘Shaping knowledge’ BCTs	0.72	0.01
MARS and ‘Natural consequences’ BCTs	0.17*	0.57
MARS and ‘Comparison of behaviour’ BCTs	0.67	0.01
MARS and ‘Associations’ BCTs	0.47	0.11
MARS and ‘Repetition and substitution’ BCTs	0.40	0.18
MARS and ‘Comparison of outcomes’ BCTs	0.00	1.00
MARS and ‘Reward and threat’ BCTs	0.21	0.50
MARS and ‘Regulation’ BCTs	0.25	0.42
MARS and ‘Antecedents’ BCTs	0.20	0.52
MARS and ‘Identity’ BCTs	0.32	0.29
MARS and ‘Scheduled consequences’ BCTs	–	–
MARS and ‘Self-belief’ BCTs	0.46	0.11
MARS and ‘Covert learning’ BCTs	–	–
**Intervention Features Categories**		
MARS and Healthy eating advice, scientific basis, and safety	0.42	0.16
MARS and Food	0.44	0.14
MARS and Tracking, goal setting	0.59*	0.04
MARS and Social	0.62	0.02
MARS and Technology	0.61*	0.03
		

Note: Spearman's correlation coefficients between variables, with Pearson's correlations marked by (*).

Behaviour change techniques (BCTs), Mobile App Rating Scale (MARS).

## 4 Discussion

This study aimed to appraise the content of the most popular diet apps available on the Apple and Android platforms. We identified 13 apps and analysed the content of these apps for behaviour change techniques, intervention features, and usability (MARS).

Both free and premium versions of the 13 apps were found to contain 17 and 19.4 BCTs, with the Noom app containing the highest number of BCTs (33). A previous study of smartphone diet apps reported use of fewer BCTs (2.3) (McAleese et al., 2022), however, the difference may result from use of an earlier taxonomy containing only 26 BCTs (Abraham and Michie, 2008), which limits the comparison of our findings. Some evidence links better diet behavioural outcomes with more BCTs (Lara et al., 2014), however, others show no association (Young et al., 2019).

The most frequently coded BCTs in our study are predominantly from the ‘Goals and planning’ and ‘Feedback and monitoring’ categories. The importance of these categories are highlighted in the NICE guidelines (National Institute for Health and Care Excellence, 2014) and ranked as the two most important categories in the Michie et al. version 1 Taxonomy (Michie et al., 2013).

Certain BCTs have been linked to greater app engagement and successful dietary outcomes. The frequent use of ‘Non-specific reward’ through gamification (Suh et al., 2018) is associated with greater app engagement (Milne-Ives et al., 2023). However, rewards can impact intrinsic motivation negatively (Lewis et al., 2016b), as intrinsic motivation is best supported through targeting values, goals and identity (Mitchell et al., 2020). The BCTs ‘Habit formation’ (Ashton et al., 2020) and ‘Social support (unspecified)’ (Lara et al., 2014) have been associated with superior outcomes in dietary interventions. A less frequently coded BCT ‘Problem solving’ only found in 30.8 % of premium app versions (Lara et al., 2014) and ‘Adding objects to the environment’, not coded at all in our study (Lin et al., 2022), have also been associated with better diet outcomes.

No identity-related BCTs were coded, however, identity plays a crucial role in supporting intrinsically motivated goals and values (Mitchell et al., 2020). The alignment of new behaviours with one's identity can support long-term behaviour change (Caldwell et al., 2018).

Further potential for app developers may lie in the application of additional BCTs from the new Behaviour Change Technique Ontology, which includes 281 BCTs (Marques et al., 2025). Two new categories of BCTs, ‘Increased awareness of behaviour’ and ‘Restructure the environment’ (Marques et al., 2025) may help individuals in the initial stages of behaviour change (Cradock et al., 2021) as awareness of behaviour and its environment is considered one of the first steps towards behaviour change (Cradock et al., 2017). It is not clear which BCTs best support behaviour change initiation or maintenance or which BCTs work best together or in what order and this requires further investigation.

Subgroup analysis showed that apps with a greater number of BCTs were significantly associated with a higher MARS score, highlighting that higher quality apps contain more BCTs. The key to BCT effectiveness lies in how the user engages with the app and to what degree the individual enacts the recommended instructions and behaviours.

The free and premium versions of the included apps contained 20.9 and 21.4 intervention features, highlighting very little difference between versions. Frequently coded features in our study ‘Educational, health and lifestyle information’, ‘Recommended water consumption’, ‘Energy requirement calculation’, ‘Blog: written by app’, ‘Social networking and support…’, ‘Barcode scanner’ and ‘Gamification’ were also frequently reported elsewhere (Chen et al., 2015; Schoeppe et al., 2017). Research supports the use of gamification to increase motivation and engagement, however, increases in engagement are only short-lasting (Sardi et al., 2017). Gamification elements through streaks, rewards and leaderboard competitions are heavily used in the popular language education app Duolingo and may be potentially useful when applied in dietary apps.

‘Safety net on weight loss that can be achieved’ was only reported in 30.8 % of premium app versions, similar to 29 % of apps reported elsewhere (Chen et al., 2015). The automated feature of many apps keeps encouraging users to lose more weight even when they are already below their healthy weight range, which is of concern. The features ‘Sharing with professionals or online coaching option’ and ‘Shopping lists’ were not frequently present in the apps identified, but have been deemed effective elsewhere (Klasnja and Pratt, 2012; Dubowitz et al., 2015). The use of shopping lists are linked with higher diet quality in low-income populations (Dubowitz et al., 2015), and have been hypothesised to conserve self-regulatory energy; making the purchase of unhealthy options less likely (Dubowitz et al., 2015). With rapid advancements in Artificial Intelligence, dietary app design might benefit from technology and the use of chatbots, which have been shown to increase fruit and vegetable consumption, but were rarely coded in any of the included apps (Singh et al., 2023). Future iterations of photo and audio diet logging need to provide more reliable and accurate diet analysis to save users' time, provide accurate information and potentially improve outcomes (Schumer et al., 2018; Hales et al., 2016). Subgroup analysis further supports the use of technology as higher app usability was associated with a greater number of technology and tracking features.

The apps in this study reached a mean total MARS score of 3.8 which is greater than scores reported in youth-targeted diet and physical activity (3.6) (Schoeppe et al., 2017) or weight-management apps (3.1) (Bardus et al., 2016). Functionality and aesthetics were the highest-rated domains, which is in agreement with previous studies (Direito et al., 2014; Schoeppe et al., 2017). The lowest-scoring domain was information, as in previous studies (Direito et al., 2014; Schoeppe et al., 2017), implying that the inclusion of evidence-based information is still lacking. ‘Evidence base’ was not assessed, as no studies were published on 69.2 % of the apps, similar to others reporting this item missing in 91 % of apps (Yamamoto et al., 2022). It is important to ascertain whether apps are safe, efficacious or have achieved behavioural or clinical outcomes.

Interestingly, we used the MARS rating, which is a validated, reliable method of assessing app quality, however, the apps that scored higher in the MARS rating were not the same as those ranked highly by the App Store or Google Play. For example, Noom achieved the highest MARS rating in our study (4.3), had the highest number of BCTs (33) and has scientific evidence supporting its effectiveness (Han and Rhee, 2021), but placed only 109th in the App Store and 93rd in Google Play. Identifying and assessing high quality apps is a challenge for users, researchers and professionals as no information is available in the App Store or Google Play as to how apps are ranked. Subjective app reviews are the only app quality metric publicly available to users, however, it is unclear how the number and quality of ratings impact the overall app ranking which suggests that the process of app ranking requires much greater transparency.

Calorie and fitness trackers have been associated with eating disorder symptomatology through higher levels of eating concern and dietary restraint, potentially influencing users' well-being negatively (Simpson and Mazzeo, 2017). Weight monitoring is considered an effective method for achieving weight loss and weight loss maintenance (Butryn et al., 2007), but may negatively affect body image and psychological health, as it is associated with an increased likelihood of eating disorder behaviours such as fasting, skipping meals, excessively exercising or purging (Hahn et al., 2021). In addition, most included apps did not include a feature of the time spent on the app. **A series of recommendations for future diet app development is outlined in Appendix 2.**

Strengths of this study include the systematic, unbiased approach to app selection. Authors had no affiliation with any of the included diet apps and analysed them as users. To support validity and reliability, coding was independently performed by two coders. This was the first study to analyse both free and premium versions of the most popular diet apps. Limitations may include the authors' atypical engagement with the app which may not be reflective of typical users, however, no data are available on how people navigate diet apps. Some popular apps could have been missed due to the selection bias of Google Play and App Store.

## 5 Conclusions

This study aimed to explore the content of popular diet apps. Thirteen apps were identified containing an average of 18 BCTs with 45 different BCTs identified. Specific BCTs frequently and not frequently reported were highlighted, with some potentially important BCTs not coded. Despite the high overall use of intervention features, a key safety feature ‘Safety net on weight loss that can be achieved’ was identified in only 4/13 apps (30.8 %). Overall usability was high, although apps performed the worst in the Information domain, often lacking an evidence base. Our study identified discrepancies between the MARS score and the app ranking system in the App Store and Google Play which require further investigation. Future research is needed to establish optimal user engagement and ascertain which BCTs, intervention and safety features are appropriate and effective.

## CRediT authorship contribution statement

**Richard Pavlicek:** Writing – review & editing, Writing – original draft, Visualization, Validation, Software, Resources, Project administration, Methodology, Investigation, Funding acquisition, Formal analysis, Data curation, Conceptualization. **Kevin A. Cradock:** Writing – review & editing, Writing – original draft, Visualization, Validation, Supervision, Software, Resources, Project administration, Methodology, Investigation, Funding acquisition, Formal analysis, Data curation, Conceptualization.

## Funding

No external funding was received for this study.

## Declaration of competing interest

The authors declare that they have no known competing financial interests or personal relationships that could have appeared to influence the work reported in this paper.

## Data Availability

Data will be made available on request.
